# A history of the term “DMARD”

**DOI:** 10.1007/s10787-015-0232-5

**Published:** 2015-05-23

**Authors:** Jonas Kure Buer

**Affiliations:** Department of Social Anthropology, University of Oslo, Blindern, Postboks 1091, 0317 Oslo, Norway

**Keywords:** DMARD, SAARD, RID, DC-ART, Drug classes, Rheumatology, History, Anthropology

## Abstract

The article outlines a history of the concept of “disease-modifying antirheumatic drugs” or DMARDs—from the emergence in the 1970s of the idea of drugs with decisive long-term effects on bone erosion in rheumatoid arthritis (RA), through the consolidation and popularisation in the term DMARD in 1980s and 1990s. It then examines the usage of the terms “remission-inducing drugs” (RIDs) and “slow-acting anti-rheumatic drugs” (SAARDs), which for some years offered competition to the term DMARDs, thus underscoring the contingency of the establishment of DMARD as a word. Finally, it juxtaposes the apparently spontaneous emergence of the three terms DMARD, SAARD and RID, and the disappearance of the latter two, with a failed attempt in the early 1990s to replace these terms with the new term “disease-controlling antirheumatic treatment” (DC-ART). The analysis highlights the paradoxical qualities of the DMARD concept as robust albeit tension ridden, while playing down the role of identified individuals and overarching explanations of purpose.

## A history of the term DMARD

Drugs designated as disease-modifying antirheumatic drugs (DMARDs) were established as mainstream antirheumatic treatments at different moments in time: injectable gold in the 1930s, hydroxychloroquine in the 1950s, azathioprine in the 1960s, sulphasalazine and methotrexate in the mid-1980s, the selectively immunomodulating drugs or so-called biological DMARDs, from 1998 on. The histories of the different drugs have been well documented.^1^ Yet the category that came to contain these drugs emerged at a separate moment in the midst of it all. Like all categories and concepts with which people equip themselves, it not only helps thinking and talking, but also fundamentally circumscribes and guides these endeavours, making certain thoughts thinkable and others unthinkable. The way in which the category of DMARDs operates, in rheumatology as in inflammopharmacology, therefore has profound implications. Even so, little has been written about its development and history. This article shall contribute to fill that void.

### The source of the Nile or its course

At the Norwegian hospital where in 2012 I did ethnographic field research, the term DMARD seemed to be taken for granted and used rather as a natural category, with little said or done implying that it had a history. As written sources could say little about its origin, I once asked the chief consultant if she knew how the term had appeared:

I think it is really just a result of some so-called experts who have been sitting and talking and needed a name for it, and then realised that, well, perhaps that was a suitable name.

That did not seem an unlikely explanation, and looking for those hypothetical experts and the place, they might have been sitting and talking, provided a point of departure for the analysis that follows. I collected testimonies from several authors of publications where I could document early usage of the term, and fragment by fragment, contours of an early history of the term could be pieced together: the purpose of introducing a concept to describe drugs that might be capable of altering the long-term course of the disease had been to distinguish these drugs from the NSAIDs, which were known only to affect the symptoms. This boundary making had remained the main function of the term throughout the late 1970s and early 1980s when many pharmaceutical companies, often with separate discovery programmes for NSAIDs and DMARDs, were working to create and develop new drugs. Any one particular moment for the birth of the concept did, however, evade me, and one source’s suggestion that the concept appeared with the introduction of penicillamine has not been possible to verify.

One need not always, however, push hard for an individual progenitor or for critical contexts and moments of creation: there may be none. Even where an individual progenitor can be singled out, as in the case of Whitehouse and the concept of “non-steroidal anti-inflammatory drugs” (see Buer 2014), and where information about the progenitor’s motivation for coining the term is available, this is of only limited use for a broader analysis, as the decisive mechanism behind the widespread use of a term like DMARD cannot be of one expert’s judgement or decision alone, but must lie in the repeated judgments and decisions of a range of different social actors. Following Barth (1990), our approach here will therefore be to see the development of the term as an “inadvertent, cumulative effect of activity to which actors [have been] propelled by perceived necessities or advantages attaching to other aspects of their activities”, i.e. to see the emergence of the concept as originating from the repeated choices of a multitude of actors to use the term, not because they wanted such a term to emerge or gain currency and not because of sympathy with the intentions of the term’s unknown, hypothetical progenitor, but because the circumstances in which all these actors acted and their immediate concerns made the term appear as purposeful.

### Considerations of method

In order to create a more precise sketch of the development of the term DMARD, I proceeded (as with the term NSAIDs, cf. Buer 2014) by means of searches in the PubMed databases for examples of early use.^2^ This approach does have certain evident shortcomings. First, the database does not contain full-text articles, consequently appearances of an item in the body text of the publications is not searchable. Also the database is not comprehensive; many older or otherwise marginal publications have not been registered. A PubMed search will thus only be able to identify use in publications which have been registered and where the sought-for term appears not in the body text of the publication, but in its title, its list of keywords, etc. Second, sought-for terms may well have been coined in a different context than that of research publishing and had their use established in those other contexts well before they started appearing in scientific journals. They may have emerged and found their shape in private thought and collegial discussions, at hospitals and universities and in pharmaceutical companies, and then, by haphazard or by gradual progression, they may have come to be perceived as useful for expressing certain ideas in public discussions, at symposia or conferences—and ultimately in scientific articles. A first mention of a term in an academic publication is thus only a hint of the term’s history, and an appearance of the phrase in a PubMed search may merely be the first searchable appearance of the phrase in PubMed. It is therefore necessary to consider the findings as indicative rather than evidential.

Even so, the data generated by the PubMed searches that I performed do tell a story about the use and development of the term. As we shall see, they tell a story of several terms competing to describe the same drugs and of one term ultimately supplanting the others. The data also reveal that the development of this term followed the same phases of development as the concept of NSAIDs had done some years earlier (see Buer 2014): the idea of drugs capable of preventing bone erosion in RA first developed in malleable descriptive phrases giving shape to the concept of “disease-modifying” drugs. The phrase then found a solid shape as a concept-category expressed by a fixed formula: “disease-modifying anti-rheumatic drugs”. From that formula spun an initialism (i.e. an abbreviation formed from initial letters), “DMARD”, which by time got established as an acronym, thus becoming word in its own right. The data trace the contours of a development where drug categories destined to last emerged as such in the day-to-day struggles of researchers, manufacturers, salesmen, physicians, bureaucrats and patients to conceptualise and put into words the fragile and sometimes conflicting connections between disease and drug, cause and effect, and hope and reality.

### The phrase “disease-modifying”

**Fig. 1:**
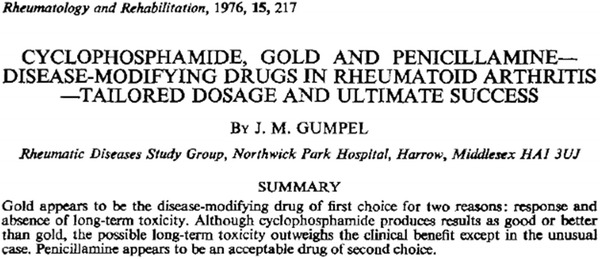
Earliest appearance of the expression “disease-modifying” in the PubMed database. With permission from Gumpel (1976), Rheumatology, Oxford University Press

The earliest use of the term “disease-modifying” that the PubMed (“all fields”) search identified was the article “Cyclophosphamide, gold and penicillamine—disease-modifying drugs in rheumatoid arthritis—tailored dosage and ultimate success” (Gumpel 1976, see Fig. 1). A closer look at the usage of the phrase in that article makes it clear that that phrase was already established when Gumpel chose to use it in his article. In PubMed, however, there is meagre evidence of its use at the time. Except for an article about influenza vaccines (Jovanovic et al. 1979), I identified no other publications containing the phrase until the beginning of the 1980s. Then, however, this changed. Three publications from 1980 contained the phrase “disease-modifying” in either title or abstract (Hunneyball 1980; McConkey et al. 1980; Bunch and O’Duffy 1980). Although my search revealed no publications from 1981 containing the phrase “disease-modifying”, the phrase reappeared in five publications from 1982 (van Wanghe and Dequeker 1982; Barnes 1982; Paulus 1982; Whisnant and Pelkey 1982; Rainsford 1982). From then on, the frequency increased: from 1983, 10 publications; 14 from 1984, 13 from 1985 and so on. Clearly, between 1980 and 1982, something had happened: the idea of disease-modifying drugs had gained momentum.

### Fixed phrase and abbreviation

**Fig. 2:**
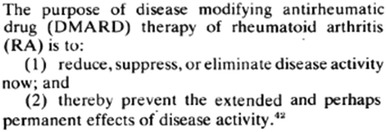
This passage from Paulus’ (1982) article is the earliest use of the initialism DMARD that I documented. Typically for initialisms, the term follows in parenthesis after the full phrase. With permission from Paulus, Annals of the Rheumatic Diseases, BMJ Publishing Group Ltd

A closer examination reveals that at approximately that time, barely preceding the sharp rise in frequency observed from 1982 on, the concept underwent two interconnected transformations which resulted in a cementation of the term: first, the idea of disease-modifying drugs ceased to be expressed in varying descriptive phrases open to reformulation and became fixed as formula, the concept-phrase “disease-modifying antirheumatic drugs”. Second, this fixed concept-phrase engendered an initialism: “DMARD”. Both transformations are tangible in the juxtaposition of the article “An overview of benefit/risk of disease-modifying treatment of rheumatoid arthritis as of today” (Paulus 1982), which is the earliest appearance of the initialism DMARD that I have documented, and the review “Recent developments in disease-modifying antirheumatic drugs” (Hunneyball 1980) published only 2 years earlier. In Hunneyball’s text, from 1980 the phrase that he employed to refer to the category still seemed malleable: while he used the phrase “disease-modifying antirheumatic drugs” in the title, he used the phrase “non-steroidal disease-modifying drugs” in the body text.^3^ Contrasting this dynamic play of semantics in Paulus text, published 2 years later, the phrase appears as fixed in the shape it retains today. It was from this fixed phrase that the initialism DMARD spun. Yet, among the numerous initialisms in Hunneyball’s text, the initialism DMARD did not figure; the malleability which the phrase of origin still retained did not allow for that. In Paulus text, by contrast, the initialism DMARD is aptly used in replacement of the full phrase (see Fig. 2).

### DMARD as a word

The next decisive change that we can observe from the PubMed search data is the establishment of the initialism DMARD as an acronym, i.e. as a proper word developed along similar lines as those the word “laser” followed, shooting off from the phrase “light amplification by stimulated emission of radiation”. This transition happened gradually, and any attempt to locate it to a fixed date risks being both futile and misleading. Nevertheless, as this development necessitated that the users of the initialism DMARD had become so familiar with its meaning that the original phrase became superfluous, the leap to wordhood can be observed in textual evidence when the phrase “disease-modifying antirheumatic drug” ceases to precede the initialism, and the term “DMARD” starts to figure independently from the concept-phrase. Further, the appearance of the stand-alone acronym in the title of scientific publications is a good indication as to when such use had become commonplace.

The earliest example of a title containing the term DMARD that I documented was a publication in Danish: “Behandling af patienter med reumatoid arthritis med DMARD (disease modifying anti-rheumatic drugs)” (Halberg 1984). In its title, the full phrase figured together with the acronym, albeit in parenthesis and in reverse order to that which would have been the case with an initialism. This use indicates that DMARD was about to be established as a word but also that the acronym still was considered to require exegesis—at least in the Danish context. The fact that that publication was the only one from the 1980s which has the isolated acronym “DMARD” in its title may indicate that Halberg’s use was somewhat premature or precursory.

**Fig. 3:**
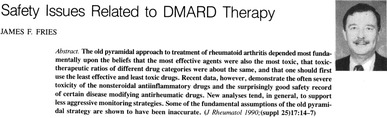
Fries, title page, first title containing acronym DMARD. With permission from Fries (1990), The Journal of Rheumatology

The next publication in which title the term DMARD figures as acronym marks a more significant beginning. This was the article “Safety issues related to DMARD therapy” (Fries 1990, see Fig. 3), which was followed shortly by “Occurrence of neoplasia in patients with rheumatoid arthritis enrolled in a DMARD Registry” (Matteson et al. 1991). From that moment and throughout the 1990s and 2000s, the use of the acronym DMARD in the titles of scientific publications multiplied. A PubMed search on 17.12.2014 identified 273 such titles. A Google search for the word^4^ gave 475,000 results.

The term DMARD had gained currency as a word. The term DMARD had been chosen again and again to create this effect, which implies that for some reason this term was perceived to best answer the cognitive and communicative challenges of the most numerous or the most influential social actors. A comparison with the development of two other terms which competed to describe the same category of drugs reveals the contingency of that development.

### Competing terms: RID and SAARD

The term DMARD was but one of the several terms, each highlighting different properties, which competed to describe the same drugs. It was the term that gained currency the fastest and the term that remained in use while the alternatives did not.

One concept which was introduced to categorise the same drugs as did DMARDs was “remission-inducing drugs” or RIDs.^5^ An “all fields” PubMed search for “remission-inducing drugs” (on 28 November 2014) gave a list of 53 texts, published between 1980 and 2010, 42 of which pertained to arthritis. Out of these 42, only eight publications, published between 1980 (Anastassiades 1980) and 1987 (Hansen et al. 1987), had the term “remission-inducing drug” in their title, and the initialism “RID” figured in only one title, but not as a proper word; RID appeared in parentheses following the full phrase “remission-inducing drugs”, a use which is indicative of status as initialism. No publications had “RID” as a stand-alone acronym in its title.

**Fig. 4:**
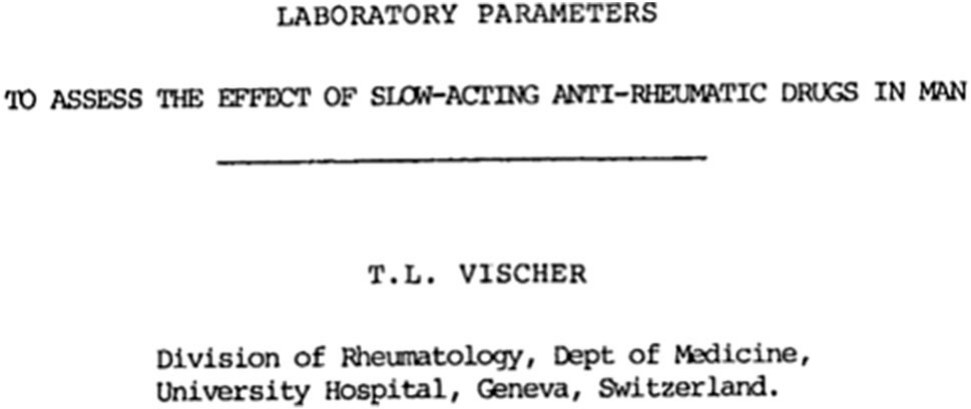
Earliest use of the term “slow-acting anti-rheumatic drugs” identified in PubMed. With permission from Vischer (1979), Agents Actions Suppl, Springer Science+Business Media

A second competing term was “slow-acting anti-rheumatic drugs” or SAARDs. PubMed searches revealed 47 publications that figured the phrase “slow-acting antirheumatic” or “slow-acting anti-rheumatic” in their title. The earliest article identified in the search was “To assess the effect of slow-acting anti-rheumatic drugs in man” (Vischer 1979, see Fig. 4), followed by “Slow-acting antirheumatic drugs” (Mowat 1982). The last publication identified was “New treatments for rheumatoid arthritis. Available and upcoming slow-acting antirheumatic drugs” (Fye 1999).^6^ After 1999, no authored publications in English registered in the PubMed database had the phrase “slow-acting antirheumatic drug” in its title.

**Fig. 5:**
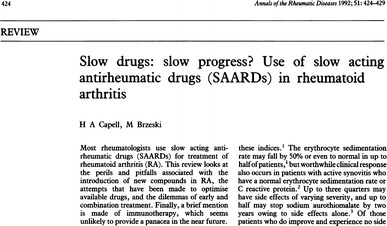
Title page of one of the three publications, in PubMed that had the initialism SAARD in their title. With permission from Capell and Brzeski (1992), Annals of the Rheumatic Diseases, BMJ Publishing Group Ltd

As for the term’s initialism (SAARD), it figured in the title of three publications (Danis et al. 1991; Capell and Brzeski 1992; van Gestel et al. 1997). In all three cases, the term figured in parenthesis following the full phrase “slow-acting anti-rheumatic drugs”, indicative of use as initialism; it was not standing alone as an independent word, as the term DMARD had come to do (Fig. 5).

Judging from these data, neither “RID” nor “SAARD” attained wordhood like “DMARD” did. The phrase “remission-inducing drugs” never even became a robust concept; it survived for some years as a standardised descriptive phrase, but went out of use as it became evident that the drugs did not induce remission (see Capell and Brzeski 1992; Scott et al. 1987). The term “slow-acting anti-rheumatic drugs” gained a more widespread use. Yet it was less popular and less robust than the term DMARD, and it did not develop as far towards becoming a word, as testified by the absence of titles with the stand-alone acronym SAARD in PubMed. Also, for both “RID” and “SAARD”, their usage, as indicated by their appearance in titles in PubMed, fell within limited time frames (1980–1987 for RID, 1979–1999 for SAARD).

### An effort to replace the term

In the early 1990s, more than a decade had passed since the use of the term “disease-modifying antirheumatic drug” had become established. The 4th joint meeting of the WHO/ILAR^7^ Task Force on Rheumatic Diseases proposed to replace the terms DMARD and SAARD. The proposed new classifications were announced in a piece co-authored by three professors of medicine from USA, UK and Australia (Paulus et al. 1992), giving broad geographical mooring.

According to the authors, the only characteristic that it had been demonstrated that the drugs referred to as SAARDs or DMARDs shared, in addition to their being “somewhat slower acting”, was their ability to provide symptomatic relief in RA (Edmonds et al. 1993b). This, we recall, was precisely the effect profile that the category was used to demarcate against. The “belief”, the authors wrote, that these drugs influenced the course of the disease was based on positive reports on the effect on radiologic progression and joint damage of some of these drugs, while in fact even those data were “by no means straightforward” (Edmonds et al. 1993b). In fact, despite the considerable development of new drugs, evaluation of DMARD/SAARD treatment in RA revealed “disappointing long-term results” (Edmonds et al. 1993b).

On this ground, the authors argued that the interchangeable use of SAARDs and DMARDs to describe the drugs was inconsistent and contributed to confusion about the actual properties of the drugs (Edmonds et al. 1993b). They proposed an adaptation of the drug classes to fit facts which testing had revealed, i.e. they advocated an adaptation to a situation where disease-modifying drugs had not yet developed but were expected to do so.

### Classification proposed (1992)

**Fig. 6:**
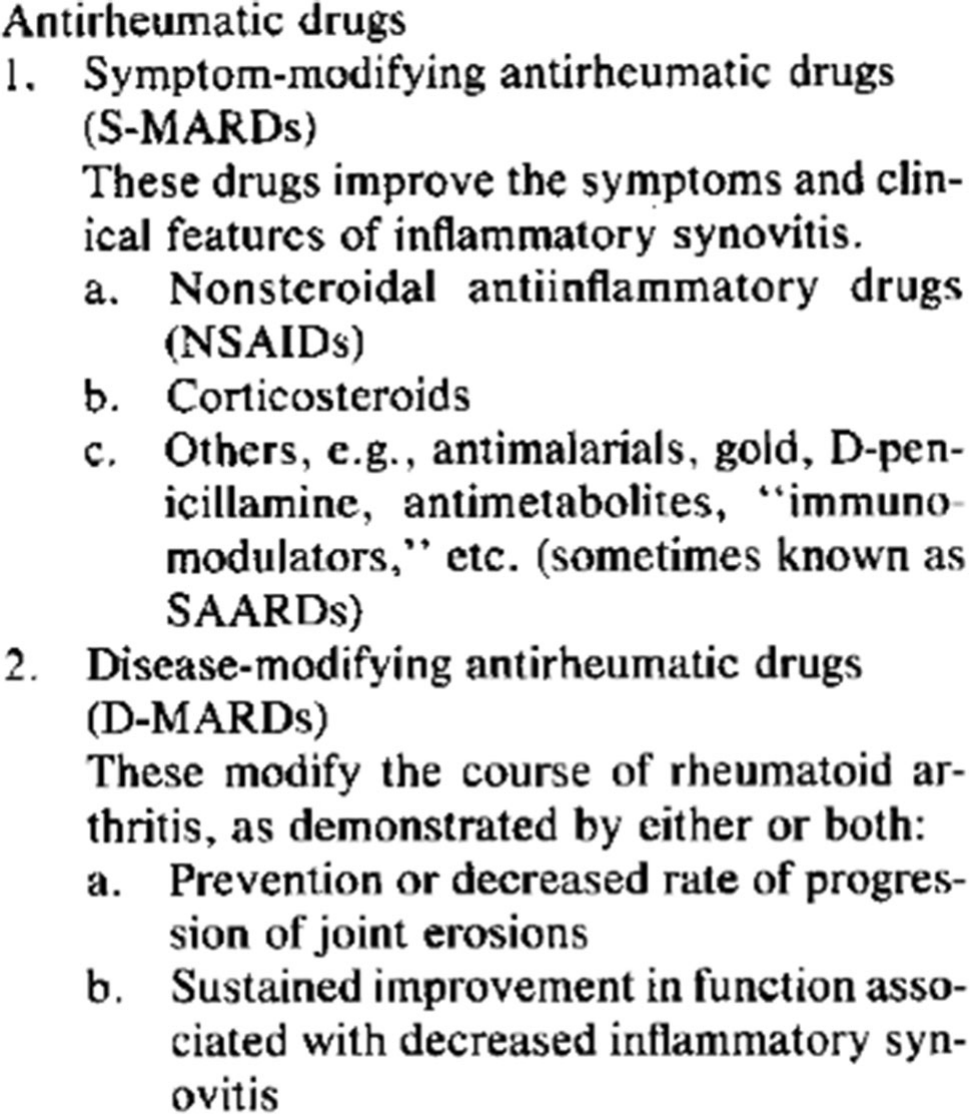
Classification of antirheumatic drugs proposed in 1992. With permission from Paulus et al. (1992), Arthritis and Rheumatism, John Wiley and Sons

The proposal introduced two new categories: symptom-modifying antirheumatic drugs (S-MARDs) and disease-modifying antirheumatic drugs (D-MARDs, see Fig. 6).

To the category, S-MARD would be assigned drug therapies that had been proven to have effect on the symptoms of the disease—i.e. pain, inflammatory activity, etc.—but which had not been proven to alter the course of the disease and prevent or delay bone destruction. The category “disease-modifying anti-rheumatic drugs” (shortened D-MARDs, with a hyphen) would comprise drugs with proven capacity to alter the course of the disease (i.e. preventing bone erosion).

### Updated proposal (1993)

**Fig. 7:**
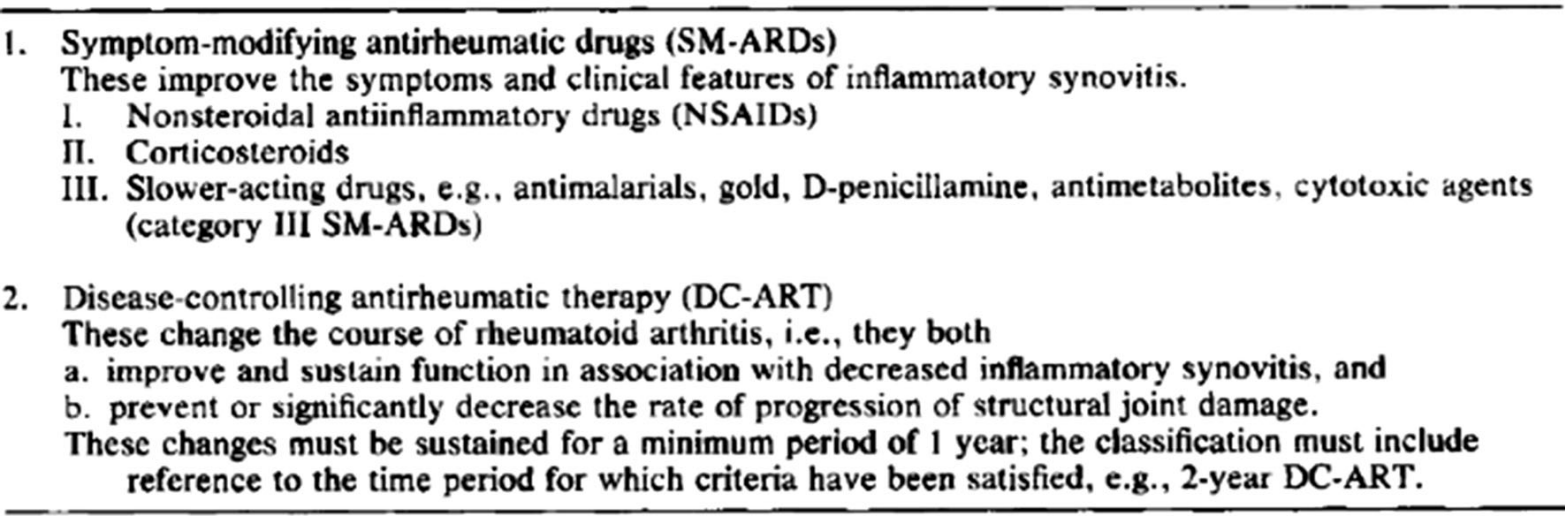
Revised proposal for classification of antirheumatic therapies. With permission from Edmonds et al. (1993b), Arthritis and Rheumatism, John Wiley and Sons

A more elaborate proposal was published the following year (Edmonds et al. 1993b). Taking into account the emergent fact that management of RA “relates as much to an overall management strategy or drug combination as to a single agent”, the most significant modification from the previous year’s proposal was the substitution of the term “D-MARD”, with the term “DC-ART” or “disease-controlling antirheumatic therapy” (see Fig. 7). This was intended to encourage research on combination treatment programs in addition to single drugs (Edmonds et al. 1993b). Taking the place of DMARDs in the classificatory system, the concept would encompass “a drug or a strategy that prevents or significantly limits anatomic damage and maintains function and well-being at follow-up periods of 5, 10, 20 years, and even longer” (Edmonds et al. 1993b).

### How the new system would work

In contrast to the classification that it was intended to supplant—where any one drug belonged to one category, the new proposed classificatory system would be employed as stepwise progressive criteria against which both existing and new drugs could be measured. Any antirheumatic drug for which symptomatic effect was demonstrated would be given an initial approval as SM-ARD (Paulus et al. 1992). With a provisional status as SM-ARD, the drug could be used in the treatment of RA and over time proves its capacity to alter the course of the disease and earn the designation as “disease-controlling antirheumatic therapy”, DC-ART. As a solution to the practical problem arising from the fact that one could not know what effect a newly developed drug would have 20 or 30 years ahead, classification included gradually progressing criteria towards long term: “…the use of this classification should invariably indicate the period over which such activity has been demonstrated, e.g., 1-year DC-ART, 2-year DC-ART, 5-year DC-ART, etc.” (Edmonds et al. 1993b).

### Outcome: an empty category and devaluation of all existing DMARDs

As no existing drugs had yet been proven to have the kind of effects one had hoped the disease-modifying drugs would have, the proposed criteria thus implied that all existing drug therapies—NSAIDs, corticosteroids and even the drugs hitherto known as DMARDs, RIDs or SAARDs—would be reclassified as symptom-modifying antirheumatic drugs of categories I, II and III, respectively (Paulus et al. 1992; Edmonds et al. 1993b).

This could be seen as a setback, but the proponents of the initiative hoped that the new classification should facilitate design and implementation of clinical trials and stimulate development of new drugs (Edmonds et al. 1993b):

The proposed classification is logical and should make antirheumatic drug development and testing more efficient. (…) The stringency of the classification should encourage those concerned with drug manufacture and rheumatic disease therapy to develop new agents and new strategies that aspire to DC-ART classification.

The stepwise progression in particular, they argued, would make it easier for drug developers and manufacturers “to show that a new drug is effective” (Edmonds et al. 1993b). The other side of the coin, from the perspective of the providing part, was that it would be much harder to get a drug recognised as a DC-ART than it had been to get it accepted as a DMARD. From a health and safety perspective, one might argue that both these effects were favourable.

### Problems

There were, however, several important challenges related to the new classification of DC-ARTs, and although the system was ratified by both ILAR and WHO (Edmonds 1994a), it soon ran into problems. There were economical and practical challenges relating to trials that the proponents of the initiative had foreseen: “Trials for classification as DC-ART will be complex, time-consuming, labor-intensive, and expensive” (Edmonds et al. 1993b). Also, before tests could start, problems would have to be handled. These pertained to method and to criteria for judging the effect of drugs over time, as well as to the perceived need of stratifying patients (Edmonds et al. 1993b). Several publications followed (Edmonds et al. 1993a; Edmonds 1994b). Soon signs emerged that the initiative had got caught in the technicalities inherent in the new categories (Edmonds and Bosi Ferraz 1994):

Given the heterogeneity of rheumatoid arthritis (…), it seems likely that some therapeutic regimens will satisfy DC-ART criteria to different degrees in different strata of patients with RA. Since it entails its own problems, it is recommended that the value and feasibility of stratification be evaluated before any attempt to design a DC-ART trial.

After this, it seems that the initiative soon stalled. The authors had correctly enough foreseen that the classification would need to be promoted though close work “with the international rheumatology community, regulatory agencies, and industry, towards their acceptance and use in drug development, testing, and regulatory control” (Edmonds et al. 1993b). This did not fare as well as it was hoped. Professor John Edmonds recalled some 20 years later:^8^

Perhaps like so many other things transacted at WHO meetings, it was endorsed, published and ignored. I expect that for a new classification to have gained currency, it would have to have been taken up by powerful American groups—but that didn’t happen and the status quo was safely maintained. In any event, it was not greatly important.

I have found little evidence of later use in RA.^9^ Just as the general silence gives a hint as to the destiny of the new classification, so does the tone of one of the few mentions of the initiative in later literature: in his historical review of old and new antirheumatic drugs, John P. Case referred to the initiative as a recommendation to use “yet another descriptive term”, while adding: “…whether such a purely semantic approach to the question adds much is certainly debatable” (Case 2001a).

### Concluding remarks

Ten years prior to the WHO/ILAR meeting where the new classifications were proposed, Anastassiades had warned about the risks of unchecked introduction of new DMARDs (Anastassiades 1980):

One cannot but be concerned about the possibility of a therapeutic hydra, with a decline in popularity of one remission-inducing drug because of serious side-effects serving as an impetus for the evaluation of many other agents, whose significant toxicity might be appreciated only gradually.

Far from being a “purely semantic approach”, as case wrote in 2001, the initiative to introduce new drug categories to supplant the problematic SAARD/DMARD concept was a forceful attempt to align the medical map with a terrain which years of clinical testing had revealed and make way for stringent testing of the many toxic agents that were introduced as powerful medicines. As a conceptual reform implicitly directed at ridding rheumatology of non-efficient drugs, it was an initiative typical to the early 1990s, when the movement known as evidence-based medicine (EBM) emerged to strengthen emphasis on scientific testing of the effect of drugs [see for instance Pope (2003).

Why then did the initiative not succeed? Technical challenges, complicated procedures and high costs may have been one factor. But in medicine, complicated procedures and high costs do not alone explain why things do not happen. Lack of support from powerful groups was a factor, but rather than providing an answer, the failure of such groups to embrace the proposal begs the question of why. And this takes us back to where we started: for the categories proposed in the early 1990s to be established and used, it would necessitate that individual actors came to consider those particular concepts more useful than their alternatives, not on an elevated level of principles and policies, but in the particular contexts of their work. This, it seems, they did not. The failure of the initiative to introduce a more stringent classification and to adjust the classificatory map with the terrain that research had revealed underscores the paradoxical qualities of the DMARD concept, which survived the attempt to replace it, as both tension ridden and robust. It also illustrates how the terms used to think and talk about drugs are the results of complicated processes—processes where argument, planning and intentional action play a lesser role than one might expect, and where the pragmatic potential that each term brings into concrete clinical, scientific, regulatory or economic contexts weighes more, in the long run, than do the original intentions of those who first put each concept into words.

## Footnotes

1 In the research for this article, I have leaned on several studies (Kean et al. 1985; Case 2001a, b; Walshe 2003; Whitehouse 2005, 2011).

2 For the different terms, I performed “text word” search for publications where the term was included (on 28 November 2014).

3 Note that the inclusion of the term “non-steroidal” in the phrase works to exclude corticosteroids from the category, just as they were excluded from the category "non-steroidal anti-inflammatory drugs". The concept of DMARD eventually came to be understood as excluding steroids, albeit implicitly.

4 For DMARD or DMARDs, "any language", on 28 December 2014.

5 This term is likely to have migrated from oncology, as the earliest appearance in PubMed of the term “remission-inducing drugs” is a paper on chemotherapy in leukaemia (Perk et al. 1975).

6 The search identified two more publications after this date, disregarded in this analysis on the ground that the one had no authors (NN 2001), while the other was in French (Van Linthoudt and Gerster 2004). Similarly, one publication in German (Wollin and Menninger 1997) has been disregarded in the account on RIDs.

7 International League Against Rheumatism.

8 Personal communication (8 January 2015).

9 There is, however, some evidence of later use of this terminology in publications pertaining to the management of ankylosing spondylitis (AS): for instance, an international specialist group (ASAS), formed in 1995, published criteria for assessing short-term improvement with SM-ARDs and was in 2002 working to develop criteria for testing infliximab and etanercept as DC-ARTs (van der Heijde et al. 2002).
